# Co-morbidity and polypharmacy in Parkinson’s disease: insights from a large Scottish primary care database

**DOI:** 10.1186/s12883-017-0904-4

**Published:** 2017-07-01

**Authors:** Gary McLean, John V. Hindle, Bruce Guthrie, Stewart W. Mercer

**Affiliations:** 1 General Practice and Primary Care, Institute of Health and Wellbeing, University of Glasgow, Glasgow, G12 9LX UK; 2 Bangor University, Bangor, Gwynedd LL57 2AS UK; 3 Population Health Sciences Division, University of Dundee, Mackenzie Building, Kirsty Semple Way, Dundee, DD2 4BF UK

## Abstract

**Background:**

Parkinson’s disease is complicated by comorbidity and polypharmacy, but the extent and patterns of these are unclear. We describe comorbidity and polypharmacy in patients with and without Parkinson’s disease across 31 other physical, and seven mental health conditions.

**Methods:**

We analysed primary health-care data on 510,502 adults aged 55 and over. We generated standardised prevalence rates by age-groups, gender, and neighbourhood deprivation, then calculated age, sex and deprivation adjusted odds ratios (OR) and 95% confidence intervals (95% CI) for those with PD compared to those without, for the prevalence, and number of conditions.

**Results:**

Two thousand six hundred forty (0.5%) had Parkinson’s disease, of whom only 7.4% had no other conditions compared with 22.9% of controls (adjusted OR [aOR] 0.43, 95% 0.38–0.49). The Parkinson’s group had more conditions, with the biggest difference found for seven or more conditions (PD 12.1% vs. controls 3.9%; aOR 2.08 95% CI 1.84–2.35). 12 of the 31 physical conditions and five of the seven mental health conditions were significantly more prevalent in the PD group. 44.5% with Parkinson’s disease were on five to nine repeat prescriptions compared to 24.5% of controls (aOR 1.40; 95% CI 1.28 to 1.53) and 19.2% on ten or more compared to 6.2% of controls (aOR 1.90; 95% CI 1.68 to 2.15).

**Conclusions:**

Parkinson’s disease is associated with substantial physical and mental co-morbidity. Polypharmacy is also a significant issue due to the complex nature of the disease and associated treatments.

## Background

Parkinson disease (PD) is a chronic, progressive neurodegenerative disease characterized by slowness of movement (bradykinesia) together with at least one of rest tremor, rigidity, and postural instability [1]. The onset of PD is rare before age 50 years and there is a sharp increase in incidence after age 60 years, such that PD has been reported as the second most common chronic neurodegenerative condition in older people in Europe after Alzheimer’s Disease [1, 2] . Although estimates of its true prevalence vary [2, 3], it is thought that it is present in about 0.3% of the general population and 1% of those over 60 years with an incidence of 8–18 per 100,000 person years [4]. Ageing populations are likely to lead to large increases in the prevalence of PD with estimates in UK suggesting that it may rise by as much as 26% by 2020 and double worldwide by 2030 [5, 6].

While PD is primarily characterised by motor symptoms, it is also associated with the development of a spectrum of non-motor symptoms as well as a number of related comorbid conditions. The non-motor symptoms include autonomic dysfunction affecting the cardiovascular, genitourinary and gastrointestinal systems as well as dysfunction of the respiratory system, skin, eyes and ears [7]. There may also be significant mental health disturbance with the development of depression and anxiety, psychosis, apathy, and fatigue as well as dementia as the disease advances [8, 9]. PD may also be associated with an increased risk of falls and osteoporosis [10]. The non-motor symptoms significantly adversely impact quality of life. The complex nature of PD and its co-morbidities may be particularly challenging for both the patient and health service provider to deal with due to health services being typically organised around single conditions. This often leads to people with multiple conditions receiving uncoordinated or fragmented care [11, 12]. In addition people with multiple conditions commonly face problems with regard to polypharmacy and consequent risk of adverse drug events and issues with drug adherence [13].

A number of studies have reported the prevalence of co-morbidity in old people with PD but most have focused on single conditions and particularly mental health problems, finding high levels of depression and psychosis [14, 15]. Studies which have included multiple conditions have tended to be small in size and focused on the effect on issues such as mobility [16]. One study specifically looked at the spectrum of co-morbidity in an incident PD cohort of 197 people in the 5 years before disease onset and the subsequent 15 years compared with a matched control group using a summary co-morbidity score (Charlson Index) and the likelihood of having one or more diagnoses within each International Classification of Diseases chapter/subchapter [17]. Before the onset of PD there was no difference in the spectrum of co-morbid disorders, but after diagnosis there was greater morbidity compared with controls with the excess morbidity reflecting either the cardinal features of PD, recognized sequelae of PD, or PD complications [17]. A Dutch study in general practice assessed the levels of co-morbidity in seven neurological disorders including PD and depression. Stroke, dementia and depression were identified as the commonest co-morbidities in PD [18].

The nature of the treatment for the motor and non-motor symptoms of PD means that patients almost inevitably end up on complex medication regimes. One case control study in Taiwan analysed the drug history in a two-year period prior to PD onset [19]. The results suggested that polypharmacy was greater in people who developed PD but the detailed nature of this relationship was unclear. Co-morbid conditions which were more common in PD included dementia, stroke, depression, and alcoholism. Hyperlipidaemia was inversely associated with PD.

There is still a need to examine the detailed relationship between PD, other conditions and associated prescribing in larger populations, in order to better understand the burden of comorbidity and polypharmacy. Therefore, this paper examines the prevalence of physical and mental health co-morbidity and polypharmacy in people with and without PD in a large population cohort of people aged 55 years and over.

## Methods

We obtained data from the Primary Care Clinical Informatics Unit at the University of Aberdeen for all 510,542 patients aged 55 and over who were alive and permanently registered with one of 314 Scottish general practices on March 31, 2007 [20]. Data on the presence of PD, 31 other common chronic physical health conditions and 7 mental health conditions were extracted (listed in Appendix 1). The dataset is representative of the whole Scottish population in terms of age, sex and socioeconomic deprivation, with a more detailed explanation available elsewhere [21]. We defined PD using a set of Read Codes used by NHS Scotland Information Services Division for this purpose. Read Codes are a coded thesaurus of clinical terms used in the NHS since 1985. They provide a standard vocabulary for clinicians to record patients findings and procedures across both primary and secondary care. For the purpose of this study we defined someone as having PD if they had a Read Code recorded of F11x9 (Cerebral degeneration in Parkinson’s disease), F12..(Parkinson’s disease), F120.Paralysis agitans.,F12z. (Parkinson’s disease NOS), F1303 (Parkinsonism with orthostatic hypotension).

To control for differences between the two populations in age, gender and socioeconomic deprivation we adopted a similar approach to that undertaken in previous papers [22–24]. We generated standardised prevalence rates by age groups (55 to 64; 65 to 74; 75 to 84 and 85 and over), gender and deprivation decile using the direct method, using a bonferroni correction for multiple comparisons. In direct standardization, estimated rates are adjusted according to the frequency distribution of a standard population (in this case the control group). The standard population is partitioned into categories, called standard strata based on the age group-gender and deprivation quintile of the individual. A weighted sum of the point estimates from the standard strata is used to produce an overall point estimate for the population. From this standardised prevalence, rates are calculated for the PD and control groups. These age-gender-deprivation standardised rates were then used to calculate age, sex, and deprivation adjusted odds ratio (ORs), and 95% confidence intervals (95% CI) for the adults with PD compared to those without (controls), for the prevalence of 31 other physical conditions and seven mental health conditions as well as by the number of overall morbidities (total physical + mental conditions) and the number of physical and mental health conditions.

Data on the number of drugs authorised for repeat prescription and issued in the previous 84 days were extracted. We chose 84 days (12 weeks) to ensure that short-term prescribing for acute conditions was not included. The count of number of repeat prescription drugs includes all pharmacologically active drugs, but excludes devices, dressings and topical preparations without significant systemic effects. Socioeconomic deprivation was measured using the Carstairs deprivation score divided into quintiles (equal fifths). The Carstairs score is based on postcode of residence and is widely used in healthcare research as a measure of socioeconomic status. We used t tests to analyse differences between groups and one-way analysis of variance for differences across age groups and deprivation deciles. For all statistical analyses, a *p* < 0·05 was considered statistically significant. All analyses were performed in Stata version 13. The NHS Grampian Research Ethics Service approved the anonymous use of these data for research purposes, thefore this study did not require individual ethics approval.

## Results

### Demographics

There were 2640 (0.5% of the total population patients with a Read Code for PD recorded (Table 1). Men were over-represented in the PD group compared with controls (55.3% vs. 45.8% for controls; *p* < 0.001). Individuals with PD were on average much older (mean age 76.4 years vs. 68.2 years for controls; *p* < 0.001), with 61% aged 75 or over compared with 26.4% of controls (*p* < 0.001). There was little difference found by distributions in deprivation quintiles.

**Table 1:** Age, gender and deprivation status, PD versus controls

Variable	PD Number (%)	No PD Number (%)
Total (%)	2640 (0.5)	507,862 (99.5)
Gender (% male)	1459 (55.3)	232,602 (45.8)
Mean Age (sd)	76.4 (9.1)	68.2 (9.7)
Age group		
55–64	308 (11.7)	219,025 (43.1)
65–74	722 (27.3)	154,558 (30.4)
75–84	1084 (41.1)	98,236 (19.3)
85 and above	526 (19.9)	36,043 (7.1)
Deprivation Quintile		
Least Deprived	562 (21.3)	99,489 (19.6)
2	635 (24.0)	117,794 (23.2)
3	612 (23.2)	117,011 (23.0)
4	464 (17.6)	93,013 (18.3)
Most Deprived	367 (13.9)	80,555 (15.9)

### Comorbidities

Overall, 7.4% of individuals with PD had no other conditions compared with 22.9% of controls (OR 0.43 95% CI 0.38–0.49 (Table 2). There were significant differences between the PD and control groups, after standardising for age, sex, and deprivation for five, six and seven or more comorbidities. For example, 30.9% had five or more conditions compared with 13.2% of controls (*p* < 0.001) with the biggest difference found for seven or more conditions (PD 12.1% vs. controls 3.9%; OR 2.08 95% CI 1.84–2.35) (table 2).

**Table 2:** Prevalence and odds ratio for number and type of comorbidities (standardised by age, gender and deprivation score)

	Parkinson’s N (prevalence %) *N* = 2640 (0.5%)	No Parkinson’s N (prevalence %) *N* = 507,862 (99.5%)	Odds ratio (95% CI) (standardised by age, gender and deprivation)^b^
Total number of morbidities^a^			
None	195 (7.4)	116,358 (22.9)	0.43 (0.38–0.49)
One	376 (14.3)	113,634 (22.4)	0.81 (0.73–0.89)
Two	487 (18.5)	94,062 (18.5)	0.99 (0.90–1.09)
Three	430 (16.3)	69,525 (13.7)	1.01 (0.91–1.12)
Four	355 (12.7)	47,109 (9.3)	1.05 (0.94–1.19)
Five	291 (11.0)	29,601 (5.8)	1.38 (1.22–1.56)
Six	208 (7.8)	17,800 (3.5)	1.56 (1.35–1.81)
Seven or more	318 (12.0)	19,773 (3.9)	2.08 (1.84–2.35)
Total number of physical conditions^a^			
None	311 (11.8)	129,846 (25.6)	0.65 (0.58–0.74)
One	509 (19.3)	123,984 (24.4)	0.90 (0.82–1.00)
Two	558 (21.1)	99,187 (19.5)	1.03 (0.94–1.13)
Three	444 (16.8)	67,710 (13.3)	1.02 (0.92–1.14)
Four	327 (12.4)	41,539 (8.1)	1.10 (0.98–1.23)
Five	233 (8.8)	23,414 (4.6)	1.30 (1.13–1.47)
Six	121 (4.5)	12,078 (2.4)	1.19 (1.01–1.37)
Seven or more	137 (5.2)	10,104 (2.0)	1.52 (1.27–1.86)
Total number of mental health conditions			
None	1477 (56.0)	406,379 (80.0)	0.33 (0.30–0.36)
One	718 (27.2)	74,234 (14.6)	2.09 (1.92–2.27)
Two	376 (14.2)	23,583 (4.6)	3.08 (2.75–3.45)
Three or more	69 (2.6)	3666 (0.7)	3.14 (2.46–4.00)

^a^Excluding PD. Includes all other physical + mental conditions

^b^All differences significant at *p* < 0.001

Restricting analysis only to physical health comorbidities showed a similar trend to total number of morbidities although differences were slightly smaller. Those with PD were less likely to have no physical conditions (PD 11.8% vs. controls 25.6%; OR 0.65 95% CI 0.58–0.74) with biggest difference again found for seven or more physical conditions (PD 5.2% vs. controls 2.0%; OR 1.52 95% CI 1.27–1.86). Table 2 also shows high levels of mental health in the PD group with 44% having at least one mental health condition. People with PD were less likely to have no recorded mental health condition compared with controls (PD 56.0% vs. controls 80.0%; OR 0.33, 95% CI 0.30–0.36), and more than twice as likely to have one mental health condition (PD 27.2% vs. controls 14.6%; OR 2.09, 95% CI 1.92–2.87) than people without PD.

### Physical health individual conditions

Very high raw prevalence rates were found for people with PD for hypertension (41.1%), constipation (27.5%), coronary heart disease (25.1%) and painful conditions (21.7%). After standardisation for age, sex and deprivation, 12 out of 30 physical conditions were significantly more prevalent in the PD group relative to controls with 17 conditions showing no significant difference and two conditions (hypertension and chronic kidney disease [CKD]) significantly less prevalent (Table 3). The biggest differences after standardisation for age, sex and deprivation were for constipation (OR 3.92, 95% CI 3.57–4.31) and epilepsy (OR 1.79 95% CI 1.34–2.40).

**Table 3:** Prevalence and odds ratios for individual physical conditions (standardised by age, gender and deprivation score). Conditions are ordered by size of odds ratio (largest to smallest)

Condition	Parkinson’s N (prevalence %) *N* = 2640	No Parkinson’s N (prevalence %) *N* = 507,862	Odds ratio (95% CI) (standardised by age, gender and deprivation)^a^
Constipation	725 (27.5)	29,629 (5.8)	3.92 (3.57–4.31)
Viral hepatitis	1 (0.0)	1684 (0.0)	2.03 (0.27–14.99) *p* = 0.48
Epilepsy	47 (1.8)	5109 (1.0)	1.79 (1.34–2.40)
Multiple sclerosis	9 (0.3)	1683 (0.3)	1.53 (0.79–2.97) *p* = 0.20
Visual impairment	77 (2.9)	6297 (1.2)	1.37 (1.09–1.73)
Stroke or transient ischaemic attack	363 (13.8)	33,146 (6.5)	1.37 (1.22–1.53)
Painful condition	573 (21.7)	85,233 (16.8)	1.34 (1.22–1.47)
Irritable bowel syndrome	123 (4.6)	21,903 (4.3)	1.33 (1.11–1.60)
Cirrhosis/chronic liver disease/alcoholic liver disease	9 (0.3)	1684 (0.3)	1.27 (0.66–2.46) *p* = 0.46
Coronary heart disease	664 (25.2)	74,789 (14.7)	1.22 (1.11–1.33)
Prostate disease	155 (5.9)	13,252 (2.6)	1.25 (1.05–1.48)
Heart failure	178 (6.7)	17,193 (3.4)	1.17 (1.00–1.37) *p* = 0.04
Any new cancer in the last 5 years	267 (10.1)	32,629 (6.4)	1.16 (1.02–1.32) *p* = 0.02
Thyrotoxicosis/thyroid disorders incl. Hypothyroidism	291 (11.0)	47,874 (9.4)	1.15 (1.01–1.31)
Glaucoma	135 (5.1)	14,166 (2.8)	1.15 (0.96–1.37) *p* = 0.10
Dyspepsia	326 (12.4)	51,172 (10.1)	1.13 (1.01–1.22)
Peripheral vascular disease (PVD)	150 (5.7)	18,800 (3.7)	1.08 (0.91–1.28) *p* = 0.34
Diverticular disease	239 (9.1)	30,692 (6.0)	1.06 (0.92–1.21) *p* = 0.38
Atrial fibrillation	212 (8.0)	22,378 (4.4)	1.03 (0.89–1.18) *p* = 0.67
Diabetes	348 (13.2)	55,548 (10.9)	1.02 (0.91–1.14) *p* = 0.72
Inflammatory arthritis and related conditions incl. Gout	269 (10.2)	41,973 (8.3)	1.02 (0.90–1.16) *p* = 0.71
Inflammatory bowel disease	25 (0.9)	4787 (0.9)	1.02 (0.69–1.52) *p* = 0.89
Hearing loss	284 (10.8)	36,486 (7.2)	1.01 (0.89–1.14) *p* = 0.89
Psoriasis or eczema	26 (0.9)	4775 (0.9)	0.93 (0.63–1.08) *p* = 0.74
Chronic sinusitis	17 (0.6)	4334 (0.9)	0.91 (0.56–1.47) *p* = 0.70
Active asthma	141 (5.3)	32,307 (6.4)	0.88 (0.74–1.04) *p* = 0.14
Migraine	8 (0.3)	3697 (0.7)	0.84 (0.42–1.69) *p* = 0.64
Hypertension	1087 (41.1)	192,833 (38.0)	0.82 (0.76–0.89)
Bronchiectasis	11 (0.4)	2311 (0.5)	0.81 (0.46–1.48) *p* = 0.50
Chronic kidney disease	226 (8.6)	31,719 (6.3)	0.80 (0.70–0.91)

^a^All differences significant at *p* < 0.001 except where stated

### Mental health conditions

Table 4 shows that those with PD had significantly higher prevalence for four of the six mental health conditions with no significant difference found for alcohol misuse and anorexia or bulimia. The highest prevalence for a mental health condition was found for depression with prevalence 28.0% for those with PD compared with 12.4% of controls (OR 3.02 95%CI 2.76–3.29). The biggest difference after standardisation for age, sex and deprivation was for schizophrenia or bipolar disease (PD 3.5% vs. controls 1.0%; OR 3.77, 95% CI 3.05–4.66), followed by dementia (PD 12.2% vs. controls 2.1%; OR 3.31, 95% CI 2.91–3.77).

**Table 4:** Prevalence and odds ratios for individual mental health conditions (standardised by age, gender and deprivation score). Conditions are ordered by size of odds ratio (largest too smallest)

Condition	Parkinson’s N (prevalence %) *N* = 2640	No Parkinson’s N (prevalence %) *N* = 507,862	Odds ratio (95% CI) (standardised by age, gender and deprivation)^a^
Schizophrenia (and related non-organic psychosis) or bipolar disorder	92 (3.5)	5216 (1.0)	3.77 (3.05–4.66)
Dementia	319 (12.2)	10,873 (2.1)	3.31 (2.91–3.77)
Learning Disability	15 (0.6)	1337 (0.3)	3.06 (1.82–5.13)
Depression	738 (28.0)	62,774 (12.4)	3.02 (2.76–3.29)
Anxiety & other neurotic, stress related & somatoform disorders	431 (16.3)	32,991 (6.5)	2.32 (2.08–2.59)
Alcohol misuse	83 (3.1)	18,454 (3.6)	1.01 (0.81–1.27) *p* = 0.87
Anorexia or bulimia	5 (0.2)	1007 (0.5)	0.91 (0.38–2.21) *p* = 0.85

^a^All differences significant at *p* < 0.001 except where stated

### Polypharmacy in people with PD versus controls

People with PD were on average on more active repeat prescriptions (mean number of repeats 6.2 vs. 3.3; *p* < 0.001). Table 5 shows that only 7.2% of those with PD were not on a repeat prescription compared with 29.3% of controls (OR 0.38; 95% CI 0.32 to 0.45; *p* < 0.001). In total 73.3% with PD were on five or more repeat prescriptions with 44.5% on five to nine repeat prescriptions compared with 24.5% of controls (OR 1.40; 95% CI 1.28 to 1.53; *p* < 0.001) and 19.2% on ten or more compared with 6.2% of controls (OR 1.90; 95% CI 1.68 to 2.15; *p* < 0.001).

**Table 5:** Prevalence and odds ratios for repeat prescribing, standardised by age, gender and number of physical conditions

Number of active repeat medications^a^	Parkinson’s N (prevalence %) *N* = 2640	No Parkinson’s N (prevalence %) *N* = 507,862	Odds ratio (95% CI) Directly standardised for age, sex, and number of physical conditions^b^
None	191 (7.2)	148,615 (29.3)	0.38 (0.32 to 0.45)
One	122 (4.6)	56,729 (11.2)	0.61 (0.51 to 0.73)
Two	167 (6.3)	53,903 (10.6)	0.71 (0.60 to 0.83)
Three	230 (8.7)	48,760 (9.6)	0.91 (0.80 to 1.05) *p* = 0.23
Four	252 (9.6)	44,000 (8.7)	0.95 (0.83 to 1.08) *p* = 0.46
Five to nine	1172 (44.5)	124,619 (24.5)	1.40 (1.28 to 1.53)
Ten or more	506 (19.2)	31,236 (6.2)	1.90 (1.68 to 2.15)

^a^Authorised for repeat issue without a consultation *and* issued in the last 84 days

^b^All differences significant at *p* < 0.001 except where stated

## Discussion

This large study of a representative UK population demonstrates that people with PD are more likely to have very high levels of co-morbidity and less likely to have no co-morbid conditions than controls. The results show a higher level of both physical and mental co-morbidity compared with controls with 12 physical and 5 mental conditions being more common in PD. The results are consistent with previous studies showing that co-morbidities in PD are often related to the wide effects of the disease itself [19–24].

### Comparison with other studies

#### Physical co-morbidity

The physical conditions which showed the biggest differences in prevalence compared with controls after standardisation were constipation and epilepsy. Constipation is a feature of PD itself, may occur many years before the onset of the motor disorder [25] and may be due to the early presence of PD pathology in the neuronal plexus in the bowel [26]. There is no known causal link between epilepsy and PD and epilepsy has not previously been recorded as a common co-morbid condition in PD. It could be that some patients documented with PD have drug-induced Parkinsonism (for example due to sodium valproate) or co-morbid stroke, which was also more prevalent in PD. The increased prevalence of visual dysfunction in the PD group may reflect the complex visual dysfunctions which are now known to be common in PD [27].The increased prevalence of painful disorders, irritable bowel, prostate disorder (which could mimicked by bladder sphincter problems in PD) and dyspepsia could all feasibly be explained by the known non-motor complications of PD [8]. The increased prevalence of coronary heart disease in PD is an important finding since comorbid vascular disease, including coronary disease is known to have an adverse effect on gait and cognition even in early PD [28]. Although it is known that cardiac dysfunction is present in PD with cardiac sympathetic denervation being associated with orthostatic symptoms [29], there have been no previous reports of an increased prevalence of coronary heart disease in PD. Although hypertension and chronic kidney disease were more common in the PD group, after adjustment for age, sex and deprivation there was a lower relative prevalence of these conditions compared with controls. PD is associated with autonomic impairment with orthostatic hypotension, which is often associated with supine hypertension, which would be missed in a normal consultation setting. Supine hypertension is possibly the explanation for the increase in prevalence of stroke in PD [30]. Cerebrovascular disease may be a contributory factor to the progression of dementia and maintenance of cerebrovascular health, including managing hypertension, may offer an opportunity for reducing the cognitive decline seen in PD [31].

#### Psychiatric co-morbidity

The psychiatric conditions which had higher prevalence in the PD group included dementia, depression and anxiety and fit with the known neuropsychiatric associations and consequences of PD. Depression and anxiety are common in PD and may be persistent in a proportion of patients [32] they are also recognised premorbid features of PD before motor symptoms develop, reflecting early limbic pathology [33]. Dementia develops in over 80% of people with PD after 15–20 years [34] and is associated with the spread of PD pathology to the hippocampus and cerebral cortex and increasing age [35]. The increased prevalence of schizophrenia and learning disability may be explained by the use of psychotropic agents in these conditions causing Parkinsonism.

#### Polypharmacy

There was a significantly higher level of polypharmacy in the PD group compared with controls even after controlling for the number of conditions. This will at least partly reflect the complex nature of the treatment of PD itself, but will also reflect treatments for comorbidities. It is not uncommon for patients to receive combinations of three or more medications for the motor disorder as well as additional medications for mood disorders, psychosis and dementia. A systematic review analysed the reasons behind poor medication adherence in PD found that drug regimen complexity and polypharmacy were among the factors associated with poor adherence [36].

#### Strengths and weaknesses

Strengths of this study include the large sample, which is representative of the Scottish population derived from 314 primary care practices. Such a large sample of ‘real world’ patients may have some advantages over a classic epidemiological study, which would almost certainly be much smaller and less representative. Using Read Codes to identify both the index condition (PD) and multiple medical comorbidities represents a trade-off between diagnostic accuracy and real-world representativeness. Estimates for some conditions may have been prone to bias. Finally, as a cross-sectional study we cannot make any inferences about casual relationships.

#### Implications for policy and practice

The co-occurrence of PD with dementia and depression significantly increases the chance of mortality [37]. The presence of co-morbidity, particularly psychiatric morbidity emphasises the importance of collaborative multidisciplinary care between primary care and secondary care services including mental health services. One small UK study looked at the impact of comorbidity in PD on consultations in primary care over 1 year and found that the majority of the consultations were for comorbid conditions rather than PD itself, which was largely seen in secondary care clinics [38]. Comorbidity was associated with a higher number of consultations and home visits [39]. The significant polypharmacy seen in PD also offers a challenge particularly in the presence of co-morbidity, advancing disease with increased mortality, requiring prioritisation of medications linked with the patient’s own priorities.

## Conclusions

This study confirms that PD is associated with significant physical and mental co-morbidity much of which is likely to be due to the complex nature of the disease itself. Polypharmacy is a significant issue in PD due to the complex nature of the treatments to control motor function as well as additional treatments used for the associated mental health disorders. The relative imbalance between the increased prevalence of coronary disease and lower prevalence of hypertension in this study requires further study since vascular factors may have a significant role in the progression of PD to dementia. The complex nature of co-morbidity and polypharmacy in PD require services to be well co-ordinated with the patient’s wishes central to complex decision making as the condition advances.
